# Baseline and stress-induced corticosterone levels across birds and reptiles do not reflect urbanization levels

**DOI:** 10.1093/conphys/coz110

**Published:** 2020-01-27

**Authors:** Allison S Injaian, Clinton D Francis, Jenny Q Ouyang, Davide M Dominoni, Jeremy W Donald, Matthew J Fuxjager, Wolfgang Goymann, Michaela Hau, Jerry F Husak, Michele A Johnson, Bonnie K Kircher, Rosemary Knapp, Lynn B Martin, Eliot T Miller, Laura A Schoenle, Tony D Williams, Maren N Vitousek

**Affiliations:** 1 Department of Ecology and Evolutionary Biology, Cornell University, Ithaca NY 14853, USA; 2 Center for Conservation Bioacoustics, Cornell Lab of Ornithology, Ithaca NY 14850, USA; 3 Biological Sciences Department, California Polytechnic State University, San Luis Obispo, CA 93407, USA; 4 Department of Biology, University of Nevada, Reno, NV 89557, USA; 5 Department of Animal Ecology, Netherlands Institute of Ecology, Wageningen, Netherlands; 6 Institute of Biodiversity, Animal Health and Comparative Medicine, University of Glasgow, Glasgow G12 8QQ, UK; 7 Coates Library, Trinity University, San Antonio, TX 78212, USA; 8 Department of Ecology and Evolutionary Biology, Brown University, Providence RI 02912, USA; 9 Max Planck Institute for Ornithology, Seewiesen 82319, Germany; 10 University of Konstanz, 78457 Konstanz, Germany; 11 Department of Biology, University of St. Thomas, St. Paul, MN 55105, USA; 12 Department of Biology, Trinity University, San Antonio, TX 78212, USA; 13 Department of Biology, University of Florida, Gainesville, FL 32608, USA; 14 Department of Biology, University of Oklahoma, Norman, OK 73019, USA; 15 Department of Global Health, University of South Florida, Tampa, FL 33620, USA; 16 Cornell Lab of Ornithology, Ithaca NY 14850, USA; 17 Office of Undergraduate Biology, Cornell University, Ithaca NY 14853, USA; 18 Department of Biological Sciences, Simon Fraser University, Burnaby, BC V5A 1S6, Canada

**Keywords:** anthropogenic noise, artificial light at night, glucocorticoid, human footprint, population density, stress

## Abstract

Rates of human-induced environmental change continue increasing with human population size, potentially altering animal physiology and negatively affecting wildlife. Researchers often use glucocorticoid concentrations (hormones that can be associated with stressors) to gauge the impact of anthropogenic factors (e.g. urbanization, noise and light pollution). Yet, no general relationships between human-induced environmental change and glucocorticoids have emerged. Given the number of recent studies reporting baseline and stress-induced corticosterone (the primary glucocorticoid in birds and reptiles) concentrations worldwide, it is now possible to conduct large-scale comparative analyses to test for general associations between disturbance and baseline and stress-induced corticosterone across species. Additionally, we can control for factors that may influence context, such as life history stage, environmental conditions and urban adaptability of a species. Here, we take a phylogenetically informed approach and use data from HormoneBase to test if baseline and stress-induced corticosterone are valid indicators of exposure to human footprint index, human population density, anthropogenic noise and artificial light at night in birds and reptiles. Our results show a negative relationship between anthropogenic noise and baseline corticosterone for birds characterized as urban avoiders. While our results potentially indicate that urban avoiders are more sensitive to noise than other species, overall our study suggests that the relationship between human-induced environmental change and corticosterone varies across species and contexts; we found no general relationship between human impacts and baseline and stress-induced corticosterone in birds, nor baseline corticosterone in reptiles. Therefore, it should not be assumed that high or low levels of exposure to human-induced environmental change are associated with high or low corticosterone levels, respectively, or that closely related species, or even individuals, will respond similarly. Moving forward, measuring alternative physiological traits alongside reproductive success, health and survival may provide context to better understand the potential negative effects of human-induced environmental change.

## Teaser

Human-induced environmental change can disturb animals, reducing individual and population health. We found no general relationship between urbanization and hormones associated with stressors across birds and reptiles, suggesting hormonal responses to disturbance vary across species and even individuals. Information beyond hormone levels is needed to inform conservation efforts.

## Introduction

Over the past decade, much research has focused on human impacts on wildlife due to rates of human population growth and increased infrastructure (Benítez-López *et al.*, 2010). Human impacts are not restricted to urban areas; animals living in otherwise undisturbed habitats may be exposed to artificial light at night (hereafter ‘ALAN’) and anthropogenic noise. Indeed, 88% of the land area in Europe and almost half of the land area in the US experience ALAN (Falchi *et al.*, 2016a), with even rural areas exposed to lights from agricultural and industrial buildings (Bennie *et al.*, 2014). Furthermore, 83% of the USA is within 1 km of a road (Riitters and Wickham, 2003), and anthropogenic noise sources have doubled ambient sound levels even in the most protected habitats in the USA (Buxton *et al.*, 2017). Fields such as conservation physiology can help predict animals’ responses to human-induced environmental change and increase the effectiveness of conservation management (Madliger *et al*., 2018).

Researchers often use glucocorticoids (hormones that can be associated with stressors) to gauge the impact of disturbance in free-living organisms. Baseline glucocorticoid levels reflect concentrations prior to the disturbance of sampling; whereas stress-induced glucocorticoid levels reflect the response to an acute stressor, such as standardized capture and restraint protocols. Both baseline and stress-induced glucocorticoid levels can be taken with relative ease in the field and have enabled conservation practitioners to take preventative action in certain cases (Tarlow and Blumstein, 2007; Busch and Hayward, 2009). For example, the impact of reduced habitat availability for common toads (*Bufo bufo*) is evident through measures of glucocorticoid concentrations at small spatial scales, whereas measures such as toad abundance can only detect impacts at larger spatial scales (Janin *et al.*, 2011). Yet, using glucocorticoids to diagnose populations that are negatively affected by human-induced environmental change remains generally challenging for two main reasons: (i) studies within and between species have found varying results with regards to the effects of human-induced environmental change on glucocorticoid levels (Table 1) and (ii) interpretations of increased baseline and/or stress-induced glucocorticoid levels differ throughout the literature (Wingfield and Kitaysky, 2002; MacDougall-Shackleton *et al.*, 2019).

**Table 1:** A comprehensive review of empirical work to date on the relationship between human-induced environmental change and corticosterone in birds and reptiles. Results for baseline cort, stress-induced cort and stress response (stress-induced minus baseline) are included for studies that investigated adults (studies on juveniles are not included). The ‘result’ column (decreased/increased/no change) refers to birds exposed to human-induced environmental change, as compared to non-disturbed birds

**Species**	**Disturbance type**	**Cort measure**	**Result**	**Reference**
Ash-throated flycatcher (*Myiarchus cinerascens*)	Noise (natural gas compressor)	Baseline	Decreased in females	Kleist *et al.* (2018)
Eastern bluebird (*Sialia sialis*)	Noise (natural gas compressor)	Baseline	Decreased in females	Kleist *et al.* (2018)
Mountain bluebird (*Sialia currucoides*)	Noise (natural gas compressor)	Baseline	Decreased in females	Kleist *et al.* (2018)
European starling (*Sturnus vulgaris*)	Noise (tramway)	Baseline	Increased in males and females	Russ *et al.* (2015)
House wren (*Troglodytes aedon*)	Noise (traffic)	Baseline	Increased in rural, but not urban, males and females	Davies *et al.* (2017)
European starling (*Sturnus vulgaris*)	Noise (traffic)	Baseline	No change in males or female	Russ *et al.* (2015)
Tree swallow (*Tachycineta bicolor*)	Noise (traffic)	Baseline	No change in females	Injaian *et al.* (2018)
Zebra finch (*Taeniopygia guttata*)	Noise (traffic)	Baseline	No change in males or females	Potvin and MacDougall-Shackleton (2015)
Tree swallow (*Tachycineta bicolor*)	Noise (traffic)	Stress-induced	Increased in females	Injaian *et al.* (2018)
House wren (*Troglodytes aedon*)	Noise (traffic)	Stress-induced	No change in rural or urban males and females	Davies *et al.* (2017)
Painted turtle (*Chrysemys picta*)	Roadway	Baseline	No change in males or females	Baxter-Gilbert *et al.* (2014)
European starling (*Sturnus vulgaris*)	ALAN	Baseline	Increased in males and females	Russ *et al.* (2015)
Great tit (*Parus major*)	ALAN	Baseline	Increased in males and females	Ouyang *et al.* (2015)
Zebra finch (*Taeniopygia guttata*)	ALAN	Baseline	Increased in males and females	Alaasam *et al.* (2018)
House finch (*Haemorhous mexicanus)*	Human presence	Baseline	Increased in urban and rural (less so) males and females	Weaver *et al.* (2018)
Marine iguana (*Amblyrhynchus cristatus*)	Human presence	Baseline	Increased in males, no change in females	French *et al.* (2017)
Painted turtle (*Chrysemys picta*)	Human presence	Baseline	No change in males or females	Polich (2016)

**Table 1:** A comprehensive review of empirical work to date on the relationship between human-induced environmental change and corticosterone in birds and reptiles. Results for baseline cort, stress-induced cort and stress response (stress-induced minus baseline) are included for studies that investigated adults (studies on juveniles are not included). The ‘result’ column (decreased/increased/no change) refers to birds exposed to human-induced environmental change, as compared to non-disturbed birds

**Species**	**Disturbance type**	**Cort measure**	**Result**	**Reference**
Painted turtle (*Chrysemys picta*)	Human presence	Stress response	No change in males or females	Polich (2016)
Ornate tree lizard (*Urosaurus ornatus*)	Urbanization	Baseline	Decreased in males and females	French *et al.* (2008)
American kestrel (*Falco sparverius*)	Urbanization	Baseline	Increased in females, but not males	Strasser and Heath (2013)
House wren (*Troglodytes aedon*)	Urbanization	Baseline	Increased in males and females	Davies *et al.* (2017)
Song sparrow (*Melospiza melodia*)	Urbanization	Baseline	Increased in males and females (only in certain years)	Foltz *et al.* (2015)
Tree sparrow (*Spizella arborea*)	Urbanization	Baseline	Increased in males and females	Zhang *et al.* (2011)
Abert’s towhee (*Pipilo aberti*)	Urbanization	Baseline	No change in males	Fokidis *et al.* (2009, 2011)
Common side-blotched lizard (*Uta stansburiana*)	Urbanization	Baseline	No change in males and females	Lucas and French (2012)
Copperhead (*Agkistrodon contortrix)*	Urbanization	Baseline	No change in males or females	Owen *et al.* (2014)
Curve-billed thrashers (*Toxostoma curvirostre*)	Urbanization	Baseline	No change in males	Fokidis *et al.* (2009, 2011)
Dark-eyed junco (*Junco hyemalis*)	Urbanization	Baseline	No change in females	Atwell *et al.* (2012)
European blackbird (*Turdus merula*)	Urbanization	Baseline	No change in males or females	Partecke *et al.* (2006)
House sparrow (*Passer domesticus*)	Urbanization	Baseline	No change in males	Fokidis *et al.* (2009)
Northern mockingbird (*Mimus poluglottos*)	Urbanization	Baseline	No change in males	Fokidis *et al.* (2009)
Song sparrow (*Melospiza melodia*)	Urbanization	Baseline	No change in males	Grunst *et al.* (2014)
Dark-eyed junco (*Junco hyemalis*)	Urbanization	Stress-induced	Decreased in males and females	Atwell *et al.* (2012)
Ornate tree lizard (*Urosaurus ornatus*)	Urbanization	Stress-induced	Decreased in males or females	French *et al.* (2008)

**Table 1:** A comprehensive review of empirical work to date on the relationship between human-induced environmental change and corticosterone in birds and reptiles. Results for baseline cort, stress-induced cort and stress response (stress-induced minus baseline) are included for studies that investigated adults (studies on juveniles are not included). The ‘result’ column (decreased/increased/no change) refers to birds exposed to human-induced environmental change, as compared to non-disturbed birds

**Species**	**Disturbance type**	**Cort measure**	**Result**	**Reference**
Song sparrow (*Melospiza melodia*)	Urbanization	Stress-induced	Decreased in males	Grunst *et al.* (2014)
Abert’s towhee (*Pipilo aberti*)	Urbanization	Stress-induced	Increased in males, depending on life history stage	Fokidis *et al.* (2009)
Curve-billed thrashers (*Toxostoma curvirostre*)	Urbanization	Stress-induced	Increased in males, depending on life history stage	Fokidis *et al.* (2009)
House sparrow (*Passer domesticus*)	Urbanization	Stress-induced	Increased in males, depending on life history stage	Fokidis *et al.* (2009)
Northern mockingbird (*Mimus poluglottos*)	Urbanization	Stress-induced	Increased in males, depending on life history stage	Fokidis *et al.* (2009)
Song sparrow (*Melospiza melodia*)	Urbanization	Stress-induced	Increased in males and females (only in certain years)	Foltz *et al.* (2015)
Curve billed thrasher (*Toxostoma curvirostre*)	Urbanization	Stress-induced	No change in males	Fokidis *et al.* (2011)
Abert’s towhees (*Pipilo aberti*)	Urbanization	Stress-induced	No change in males	Fokidis *et al.* (2011)
Dark-eyed junco (*Junco hyemalis*)	Urbanization	Stress response	Decreased in males and females	Atwell *et al.* (2012)
Copperhead (*Agkistrodon contortrix)*	Urbanization	Stress response	Decreased in males and females	Owen *et al.* (2014)
European blackbird (*Turdus merula*)	Urbanization	Stress response	Decreased in males (winter and spring) and females (winter only)	Partecke *et al.* (2006)
Common side-blotched lizard (*Uta stansburiana*)	Urbanization	Stress response	Increased in males and females	Lucas and French (2012)

A recent study assessing a broad variety of stressors (natural and anthropogenic) found no consensus endocrine profile for chronic stress in wild animals (Dickens and Romero, 2013), thus challenging the validity of the common assumption that higher baseline or stress-induced corticosterone levels (the primary glucocorticoid in birds and reptiles, hereafter ‘cort’) indicate greater levels of disturbance and stress. Yet, stressors associated with human-induced environmental change may be functionally different than natural stressors (e.g. food availability, temperature) given their novelty on an evolutionary timescale. To date, no large-scale pattern of human-induced environmental change (e.g. urbanization, anthropogenic noise, ALAN) on glucocorticoid profiles across birds and reptiles has been identified; studies have found baseline and stress-induced cort to increase, decrease or remain the same given various exposure regimes (Table 1). It remains unknown if this lack of a pattern stems from context dependency in how disturbance affects cort (e.g. geographic locations, life history stages) or if, in fact, there is no general pattern in how animals respond physiologically to human-induced environmental change.

Further, increased baseline and stress-induced cort levels have been alternatively interpreted as an animal appropriately coping with, or being negatively affected by, a stressor (Wingfield and Kitaysky, 2002; MacDougall-Shackleton *et al.*, 2019). Differing interpretations of increased baseline and stress-induced cort are perhaps, in part, due to the fact that stressors vary in their constancy; some stressors are more acute (e.g. capture), while others are more chronic (e.g. noise exposure; Dickens and Romero, 2013). Increased stress-induced cort may be adaptive in the context of acute stressors by increasing one’s likelihood of escape. Indeed, male tree lizards (*Urosaurus ornatus*) with experimentally elevated cort concentrations showed enhanced anti-predator responses during predator encounters (Thaker *et al.*, 2009). However, chronic stressors that result in continuously elevated baseline and stress-induced cort (i.e. no acclimation or habituation) can be associated with adverse effects, such as reduced immune and reproductive function, suppressed growth and neuronal cell death across taxa (Rich and Romero, 2005; Kvamme *et al.* 2013). Additionally, physiological responses to a given stressor will likely depend on individuals’ past exposure to stressors (Monaghan and Haussmann, 2015).

Given the large number of studies that have reported cort concentrations worldwide over the last few decades, large-scale comparative analyses are now possible. Large-scale comparative analyses can test for general relationships between human-induced environmental change and cort levels across species, while controlling for life history, environmental factors and urban adaptability of a species (Blair 2001; Madliger and Love, 2015). Identifying the presence or absence of a general pattern may also help identify the contexts in which increased baseline and/or stress-induced cort levels warrant preventative conservation action. This technique was recently used to explore relationships between International Union for the Conservation of Nature listing status, location within a geographic range and cort concentrations in birds and reptiles (Martin *et al.*, 2018).

**Figure 1:**
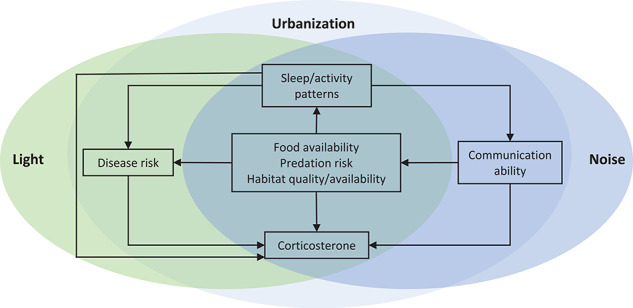
Schematic of potential pathways by which urbanization, and specifically ALAN and anthropogenic noise, can affect baseline and/or stress-induced cort in birds and reptiles.

Here, we use an established database of baseline and stress-induced cort levels across free-living vertebrates (HormoneBase.org; Vitousek *et al.*, 2018) to test multiple hypotheses regarding the relationship between baseline or stress-induced cort and large-scale patterns of human-induced environmental change, such as urbanization (as measured by human footprint index and human population density), anthropogenic noise and ALAN in birds and reptiles (Fig. 1; Bonier, 2012; Swaddle *et al.*, 2015; French *et al.*, 2018). We also test for relationships between baseline and stress-induced cort levels and exposure to human-induced environmental change for bird species with different levels of urban adaptability (e.g. urban exploiter, avoider or adapter). We account for variation in glucocorticoids due to environment (temperature and precipitation), life history stage (breeding v. non-breeding season), sex, mass and maximum number of lifetime breeding events by including these parameters in our analyses. While some of our data are relatively coarse in scale (resolution of geographic locations range from 0.5 m to 1 km, see below for details), general patterns, such as variation in average cort levels across populations should be identifiable and greater than within population variation (Addis *et al.*, 2011; Krause *et al.*, 2014; Vitousek *et al.*, 2019).

We predict that ALAN is positively related to baseline cort in birds and reptiles, given results from European blackbirds (*Turdus merula*; Russ *et al.*, 2015), zebra finches (*Taeniopygia guttata*; Alaasam *et al.*, 2018) and great tits (*Parus major*; Ouyang *et al.*, 2015). We also predict that baseline and stress-induced cort are negatively related to anthropogenic noise exposure in birds and reptiles, as chronic noise exposure has been found to limit the ability to respond to subsequent acute stressors in free-living passerines (i.e. downregulation of stress-induced corticosterone after handling; Injaian *et al*., 2018; Kleist *et al*., 2018). For both noise and light pollution, we also predict an interaction effect between urban adaptability and the disturbance parameter, such that urban avoiders will show the greatest alteration in baseline and/or stress-induced cort. It is difficult to predict whether human footprint (a measure based on infrastructure, land cover and human access to natural areas; Venter *et al.*, 2016b) and human population density will be positively or negatively associated with baseline or stress-induced cort, given that the relationship between urbanization and baseline or stress-induced cort varies in birds and reptiles (Table 1). Similarly, a lack of empirical evidence makes it difficult to form hypotheses about the association between ALAN and stress-induced cort. If our phylogenetic comparative analysis does not show a general pattern across species with regards to changes in baseline or stress-induced cort, our results would suggest that glucocorticoid levels alone cannot be used by researchers or conservation practitioners as general indicators of anthropogenic impacts. Additionally, if our results show that environmental and life history stage variables explain much of the variation in baseline or stress-induced cort, this study would support the consideration of context in studies of anthropogenic disturbances on glucocorticoids in free-living animals.

## Materials and methods

### Baseline and stress-induced cort data

We used HormoneBase (Vitousek *et al.*, 2018) to obtain data on baseline and stress-induced cort concentrations in birds and reptiles. Baseline cort measures included here were taken within 3 min of capture and stress-induced cort levels represent peak measures that were generally taken 15–60 min after capture (unless authors specifically indicated that peak cort in that population or species occurred >60 min after capture). All baseline and stress-induced cort data represent the mean concentration for each sex. Although HormoneBase includes data across vertebrate species, relatively small sample sizes in some taxa and/or the inapplicability of available disturbance metrics to aquatic taxa limited our analysis to birds and reptiles. In reptiles we included data for baseline cort only, as there were not enough data for a formal analysis of stress-induced cort in this group.

### Anthropogenic data

We used geographic location (latitude and longitude in degrees decimal) to match each cort measure with metrics of anthropogenic disturbance. Global data were available for human population density, human footprint index and ALAN, whereas data for anthropogenic noise levels were only available in the USA. Human population density was calculated by dividing population counts (acquired through national censuses and population registers from the year 2000) by land area on a 1 km grid; thus, measurements are persons/km^2^ (Center for International Earth Science Information Network (CIESIN), Columbia University, 2016). Human footprint indices were compiled using weighted measures of direct and indirect human pressures on the environment (e.g. extent of built environment, crop land, pasture land, human population density, night-time lights, railways, road and navigable waterways) at a high resolution (median = 0.5 m,) and measured on a scale from zero to 50, as described in Venter *et al.* (2016a). Human footprint indices were available from 1993 and 2009, whereas the data included in this study ranged from 1969 to 2015. For each measure, we used the human footprint index taken closest in time; thus, the metric from 1993 was used for studies that measured cort levels from 1969 to 2001, and the human footprint index from 2009 was used for studies that measured cort from 2002 to 2015. Although this method may cause human footprint indices to be over- or underestimated in geographic locations that experienced (de)urbanization in the past few decades, only 10% of locations had changes in human footprint indices >30% between 1993 and 2009. ALAN data included here were measures of sky brightness (μcd/m^2^), which were modelled using satellite measures of upward radiance from artificial sources, with a spatial resolution of 742 m (Falchi *et al.*, 2016a; Falchi *et al.*, 2016b). Data on anthropogenic noise (A-weighted L_50_ sound pressure levels dB re 20 μPa) were available from the National Park Service (NPS, 2014) and based on Random Forest models that explain the relationship between long-term measurements of ambient sound pressure level and geospatial features such as topography, climate, hydrology and anthropogenic activity (Breiman, 2001). Anthropogenic sources were isolated from models of existing soundscapes (e.g. Buxton *et al.*, 2017) through logarithmic subtraction of the natural sound levels from existing sound level estimates. Noise data had a resolution of 270 m^2^. Due to differences in available data sets, our sample sizes varied between models (see sample sizes listed in Tables 2 and 3).

**Table 2:** Model comparisons for the relationship between human-induced environmental change and avian baseline and stress-induced corticosterone, using global and US-based data

**Model** ^*^	**K**	**DIC**	**ΔDIC**	**Weight**
*Global data, avian baseline (n = 487 measures from 79 species)*				
avian baseline cort ~ mass + sex + temp + precip + life history stage + max breeding attempts + life history stage^*^max breeding attempts + human footprint index + ALAN + human population density	15	693.76	0	0.162
avian baseline cort ~ mass + sex + temp + precip + life history stage + max breeding attempts + life history stage^*^max breeding attempts + ALAN	13	694.29	0.520	0.125
avian baseline cort ~ mass + sex + temp + precip + life history stage + max breeding attempts + life history stage^*^max breeding attempts + human footprint index	13	694.64	0.874	0.104
avian baseline cort ~ mass + sex + temp + precip + life history stage + max breeding attempts + life history stage^*^max breeding attempts (*null model*)	12	694.65	0.882	0.104
avian baseline cort ~ mass + sex + temp + precip + life history stage + max breeding attempts + life history stage^*^max breeding attempts + human footprint index + ALAN + urban adaptability + ALAN^*^urban adaptability	17	694.88	1.111	0.093
avian baseline cort ~ mass + sex + temp + precip + life history stage + max breeding attempts + life history stage^*^max breeding attempts + human population density	13	694.92	1.156	0.091
avian baseline cort ~ mass + sex + temp + precip + life history stage + max breeding attempts + life history stage^*^max breeding attempts + urban adaptability	14	694.98	1.215	0.088
avian baseline cort ~ mass + sex + temp + precip + life history stage + max breeding attempts + life history stage^*^max breeding attempts + human footprint index + urban adaptability + human footprint index^*^urban adaptability	17	695.04	1.274	0.085
avian baseline cort ~ mass + sex + temp + precip + life history stage + max breeding attempts + life history stage^*^max breeding attempts + human footprint index + ALAN + human population density + urban adaptability + + human footprint index^*^urban adaptability + ALAN^*^urban adaptability + human population density^*^urban adaptability	23	695.26	1.500	0.076
avian baseline cort ~ mass + sex + temp + precip + life history stage + max breeding attempts + life history stage^*^max breeding attempts + human population density + urban adaptability + human population density^*^urban adaptability	17	695.38	1.612	0.072
avian baseline cort ~ 1 (*intercept only*)	5	717.51	23.747	0
			
*Global data, avian stress-induced (n = 278 measures from 58 species)*				
avian stress-induced cort ~ baseline cort + mass + sex + life history stage + max breeding attempts + life history stage^*^max breeding attempts + urban adaptability	12	188.05	0	0.266
avian stress-induced cort ~ baseline cort + mass + sex + life history stage + max breeding attempts + life history stage^*^max breeding attempts + human footprint index + urban adaptability + human footprint index^*^urban adaptability	15	188.45	0.402	0.217
avian stress-induced cort ~ baseline cort + mass + sex + life history stage + max breeding attempts + life history stage^*^max breeding attempts (*null model*)	10	189.05	0.997	0.161
avian stress-induced cort ~ baseline cort + mass + sex + life history stage + max breeding attempts + life history stage^*^max breeding attempts + ALAN	11	190.52	2.470	0.077
avian stress-induced cort ~ baseline cort + mass + sex + life history stage + max breeding attempts + life history stage^*^max breeding attempts + human population density	11	190.61	2.559	0.074

**Table 2:** Continued

**Model** ^*^	**K**	**DIC**	**ΔDIC**	**Weight**
avian stress-induced cort ~ baseline cort + mass + sex + life history stage + max breeding attempts + life history stage^*^max breeding attempts + human footprint index + ALAN + urban adaptability + ALAN^*^urban adaptability	15	191.04	2.990	0.060
avian stress-induced cort ~ baseline cort + mass + sex + life history stage + max breeding attempts + life history stage^*^max breeding attempts + human population density + urban adaptability + human population density^*^urban adaptability	15	191.84	3.794	0.040
avian stress-induced cort ~ baseline cort + mass + sex + life history stage + max breeding attempts + life history stage^*^max breeding attempts + human footprint index + ALAN + human population density	13	192.39	4.344	0.030
avian stress-induced cort ~ mass + sex + temp + precip + life history stage + max breeding attempts + life history stage^*^max breeding attempts + human footprint index + ALAN + human population density + urban adaptability + + human footprint index^*^urban adaptability + ALAN^*^urban adaptability + human population density^*^urban adaptability	21	193.81	5.763	0.015
avian stress-induced cort ~ 1 (*intercept only*)	5	288.45	100.398	0
			
*USA data, avian baseline (n = 344 measures from 61 species)*				
avian baseline cort ~ mass + sex + temp + precip + life history stage + max breeding attempts + life history stage^*^max breeding attempts + anthropogenic noise + urban adaptability + anthropogenic noise^*^urban adaptability	17	490.07	0	0.281
avian baseline cort ~ mass + sex + temp + precip + life history stage + max breeding attempts + life history stage^*^max breeding attempts (*null model*)	12	490.19	0.125	0.264
avian baseline cort ~ mass + sex + temp + precip + life history stage + max breeding attempts + life history stage^*^max breeding attempts + anthropogenic noise	13	490.27	0.199	0.254
avian baseline cort ~ mass + sex + temp + precip + life history stage + max breeding attempts + life history stage^*^max breeding attempts + urban adaptability	14	490.74	0.669	0.201
avian baseline cort ~ 1 (*intercept only*)	5	508.21	18.143	0
			
*USA data, avian stress-induced (n = 214 measures from 45 species)*				
avian stress-induced cort ~ baseline cort + mass + sex + life history stage + max breeding attempts + life history stage^*^max breeding attempts + urban adaptability	12	132.54	0	0.421
avian stress-induced cort ~ baseline cort + mass + sex + life history stage + max breeding attempts + life history stage^*^max breeding attempts + anthropogenic noise + urban adaptability + anthropogenic noise^*^urban adaptability	15	133.42	0.883	0.271
avian stress-induced cort ~ baseline cort + mass + sex + life history stage + max breeding attempts + life history stage^*^max breeding attempts (*null model*)	10	134.18	1.64	0.185
avian stress-induced cort ~ baseline cort + mass + sex + life history stage + max breeding attempts + life history stage^*^max breeding attempts + anthropogenic noise	11	134.99	2.453	0.123
avian stress-induced cort ~ 1 (*intercept only*)	5	178.20	45.660	0

^*^All models include ‘population ID’, ‘lab ID’ and ‘species’ as random effects.

**Table 3:** Model comparisons for the relationship between human-induced environmental change and reptilian baseline corticosterone, using global and US-based data sets

**Model** ^*^	**K**	**DIC**	**ΔDIC**	**Weight**
*Global data, reptilian baseline (n = 108 measures from 32 species)*				
reptilian baseline cort ~ mass + sex + temp + precip + life history stage + max breeding attempts + life history stage^*^max breeding attempts (*null model*)	12	556.41	0	0.26
reptilian baseline cort ~ mass + sex + temp + precip + life history stage + max breeding attempts + life history stage^*^max breeding attempts + human footprint index	13	556.72	0.31	0.22
reptilian baseline cort ~ mass + sex + temp + precip + life history stage + max breeding attempts + life history stage^*^max breeding attempts + human population density	13	556.74	0.33	0.22
reptilian baseline cort ~ mass + sex + temp + precip + life history stage + max breeding attempts + life history stage^*^max breeding attempts + ALAN	13	557.26	0.95	0.17
reptilian baseline cort ~ mass + sex + temp + precip + life history stage + max breeding attempts + life history stage^*^max breeding attempts + human footprint index + ALAN + human population density	15	557.62	1.21	0.14
reptilian baseline cort ~ 1 (*intercept only*)	5	565.90	9.493	0
				
*USA data, reptilian baseline (n = 227 measures from 15 species)*				
reptilian baseline cort ~ mass + sex + temp + precip + life history stage + max breeding attempts + life history stage^*^max breeding attempts (*null model*)	12	208.11	0	0.71
reptilian baseline cort ~ mass + sex + temp + precip + life history stage + max breeding attempts + life history stage^*^max breeding attempts + anthropogenic noise	13	210.60	2.49	0.20
reptilian baseline cort ~ 1 (*intercept only*)	5	212.34	4.24	0.09

^*^All models include ‘population ID’, ‘lab ID’ and ‘species’ as random effects.

### Species-level urban adaptability

In an attempt to better understand the role that previous adaptation to disturbance has in a species’ response to urbanization, we created an ‘urban adaptability’ parameter that characterized each species as an urban avoider, urban adapter or urban exploiter. We conducted a literature review to characterize the urban adaptability of each bird species included in our analysis (Table S1). We did not classify reptiles, as there were less published data regarding abundance of reptile species included in HormoneBase along an urban:rural gradient. We assigned bird species that primarily bred in urban areas as exploiters (e.g. house sparrows, yellow-vented bulbuls, *n* = 32 samples from 2 species), birds that commonly bred along an urban:rural gradient as adapters (e.g. black-capped chickadees, barn swallows, *n* = 222 samples from 38 species) and birds that were uncommon near urbanized areas as avoiders (e.g. pine siskin, wood thrush, *n* = 233 samples from 61 species). Some bird species in HormoneBase have been documented in urban areas during the migratory period; however, their relative use of stopover sites along an urban:rural gradient is unclear. Therefore, these species were not assigned an urban adaptability and were not included in the analyses (*n* = 6). Additionally, other species did not have enough published data to confidently categorize urban adaptability and these species were also removed from the analyses (*n* = 6). For many species that bred in remote areas, there were no published data explicitly comparing abundance along an urban:rural gradient. However, we feel confident in our classification of these species as ‘avoiders’, given their non-existence near heavily human-altered landscapes. It is important to note that one limitation of this ‘urban adaptability’ parameter is that populations, and even individuals, of a given species often vary in their previous exposure to human-induced environmental change based on differences in fine-scale habitat use and/or history of urban colonization (Ouyang *et al*., 2018). Therefore, our species-level ‘urban adaptability’ parameter may not be equally accurate across individuals and populations included in our analysis.

### Phylogenetic tree

We included phylogenetic information in all models to account for the expected similarity in baseline and stress-induced cort of closely related species. Specifically, we began with the ultrametric, fully resolved phylogeny published in association with HormoneBase (Johnson *et al.*, 2018). This phylogeny was created using a time-dated backbone phylogeny from the TimeTree of Life (Kumar *et al.*, 2017), which included one tip for each of the major animal lineages included in HormoneBase, such that each row matched one tip of a lineage-specific tree. We pruned the original tree used in HormoneBase to include only the species used in our study.

### Statistical analyses

Given that we had multiple observations per species, we used the *MCMCglmm* package in R (Hadfield, 2010) to conduct phylogenetically informed analyses of the relationship between human-induced environmental change and baseline and stress-induced cort in birds, and baseline cort in reptiles. In addition to the main effects of human footprint index, human population density, ALAN and anthropogenic noise, we included an interaction term between each anthropogenic parameter and urban adaptability in avian models. We also included parameters that were previously established as important predictors of baseline and stress-induced cort (Vitousek *et al.*, 2019). Specifically, we included sex (female = 456, male = 666), mass (mean = 2577.2 g ± 21814.6 SD), maximum number of lifetime breeding attempts (mean = 24.8 attempts ±21.5 SD), life history stage (breeding = 876 or non-breeding = 246) and an interaction between maximum number of lifetime breeding attempts and life history stage, as fixed effects in all models. Data for these fixed effects were compiled from a variety of reputable sources, such as primary scientific articles, Animal Diversity Web (animaldiversity.org), Encyclopedia of Life (eol.org) and Birds of North America (birdsna.org; see Johnson *et al*., 2018 for more details). We used *Rphylopars* to impute missing data for the ‘maximum lifetime breeding attempt’ parameter based on estimations of trait covariances across and within species (~5% and 3% of data for ‘maximum lifetime breeding attempts’ were imputed for analyses of baseline and stress-induced cort in birds, respectively; ~37% of data for ‘maximum lifetime breeding attempts’ were imputed for analysis of baseline cort in reptiles; Goolsby *et al.*, 2016). Urban adaptability, sex and life history stage were set as factors in the model, with ‘urban adapter’, ‘female’ and ‘breeding’ levels set as the default levels, respectively. We also included species (the matrix of phylogenetic relatedness), population identity (based on geographic location of the study included in HormoneBase) and hormone lab identity as random effects in all models (Vitousek *et al.*, 2019). Additionally, in models of baseline cort, we used relevant temperature (monthly average of daily mean temperature, gathered on a 0.5 degree grid) and precipitation (cumulative mm per month, gathered on a 0.5 degree grid) data from the CRU-TS 4.0 Climate Database (Harris *et al.*, 2014), as described by Johnson *et al*. (2018). Finally, we included baseline cort as a fixed effect in models of stress-induced cort. We found no issues of collinearity between all parameters, which we checked by creating a correlation matrix (*ggcor* function in the *arm* package, r < 0.7 for all pairwise comparisons). We used weakly informative priors (V = 1, nu = 0.002) to rule out unreasonable parameter values. We ran each model with 1 000 000 iterations, a burn in of 5000 and a thin of 200.

We natural-log transformed hormone data and other non-normally distributed continuous variables before analyses. Additionally, we added a constant to ALAN (+1), human footprint index (+1), human population density (+1) and temperature (+22) to ensure that all values were greater than zero. All trace plots were visually inspected to check that the chains had converged and autocorrelations were calculated to ensure that each successive value in the output did not strongly depend on the previous one (Hadfield, 2010). All models were run four times to confirm the stability of the results.

We evaluated our models using deviance information criterion (DIC), which uses deviance as a measure of fit and automatically estimates a penalty for model complexity in Bayesian models (Bolker *et al.*, 2009). Our set of candidate models was comprised of single-hypothesis models that included all covariates described above, in addition to one, all or none (null model) of our anthropogenic parameters, both with and without the urban adaptability interaction term. We also compared these models to an intercept-only model to ensure that the covariates that were previously important predictors of baseline and stress-induced cort maintained their relevance, despite using a subset of the data (Vitousek *et al.*, 2019; see Tables 2 and 3 for a full model list). For the top-ranked models, we estimated the β parameter estimates and 95% credible intervals (CI) of each parameter (Table 4). We assessed the importance of parameter estimates based on whether the 95% CI overlapped zero.

**Table 4:** β estimates ±95% CIs, calculated using ±1.96 standard error (SE) for each parameter (intercept and fixed effects only) included in the top-ranked model from each analysis

**Model**	**Parameter** ^*^	**β estimate**	**CI**	**Effective sample size**
*Global data, avian baseline (n = 487 measures from 79 species)*	*(intercept)*	4.08	2.28, 5.87	4975
	mass	−0.23	−0.50, 0.03	5608
	sex (male)	0.04	−0.06, 0.14	5220
	*life history stage (non-breeding)*	−0.82	−1.38, −0.22	4719
	max breeding attempts	0.15	−0.13, 0.44	4975
	*life history stage (non-breeding)* ^*^ *max breeding attempts*	0.24	0.05, 0.43	4729
	*temperature*	−0.28	−0.49, −0.06	4975
	precipitation	−0.09	−0.25, 0.04	4975
	ALAN	−0.24	−0.58, 0.09	4975
	human footprint index	−0.05	−0.26, 0.15	5474
	human population density	0.08	−0.01, 0.16	4975
*Global data, avian stress-induced* *(n = 278measures from 58 species)*	*(intercept)*	2.21	1.50, 2.98	4876
	*baseline cort*	0.32	0.26, 0.39	4975
	*sex (male)*	0.16	0.06, 0.25	4975
	life history stage (non-breeding)	0.20	−0.29, 0.69	4975
	*max breeding attempts*	0.27	0.10, 0.44	4975
	life history stage (non-breeding)^*^max breeding attempts	−0.14	−0.30, 0.01	4975
	urban adaptability (avoid)	0.15	−0.04, 0.22	4975
	urban adaptability (exploit)	−0.14	−0.30, 0.01	3979
*US data, avian baseline (n = 344 measures from 61 species)*	*(intercept)*	3.14	0.63, 5.50	4700
	mass	−0.19	−0.55, 0.18	5425
	sex (male)	0.06	−0.05, 0.18	4975
	*life history stage (non-breeding)*	−0.87	−1.60, −0.12	5218
	max breeding attempts	0.12	−0.30, 0.52	4975
	*life history stage (non-breeding)* ^*^ *max breeding attempts*	0.30	0.07, 0.54	4975
	temperature	−0.23	−0.48, 0.01	4975
	precipitation	−0.06	−0.24, 0.13	4975
	anthropogenic noise	0.18	−0.06, 0.41	4975
	*urban adaptability (avoid)*	0.85	0.11, 1.62	4975
	urban adaptability (exploit)	0.67	−1.17, 2.49	4772
	*anthropogenic noise* ^*^ *urban adaptability (avoid)*	−0.36	−0.68, −0.03	4764
	anthropogenic noise^*^urban adaptability (exploit)	−0.13	−0.84,0.56	4975

**Table 4:** Continued

**Model**	**Parameter** ^*^	**β estimate**	**CI**	**Effective sample size**
*US data, avian stress-induced (n = 214 measures from 45 species)*	*(intercept)*	2.47	1.70, 3.19	4975
	*baseline cort*	0.27	0.19, 0.34	4975
	*sex (male)*	0.18	0.08, 0.28	5323
	life history stage (non-breeding)	0.002	−0.54, 0.55	5031
	*max breeding attempts*	0.20	0.14, 0.38	4975
	life history stage (non-breeding)^*^max breeding attempts	−0.06	−0.03, 0.35	4961
	urban adaptability (avoid)	0.15	−0.04, 0.35	5346
	urban adaptability (exploit)	−0.06	−0.22, 0.12	4961
				
*Global data, reptilian baseline (n = 108 measures from 32 species)*	(intercept)	2.56	−0.40, 5.45	4975
	mass	−0.03	−0.31, 0.22	5426
	*sex (male)*	−0.42	−0.68, −0.16	4975
	life history stage (non-breeding)	−0.78	−1.98, 0.41	4975
	max breeding attempts	−0.20	−0.73, 0.31	4975
	life history stage (non-breeding)^*^max breeding attempts	0.28	−0.08, 0.60	4975
	temperature	0.16	−0.15, 0.48	4975
	precipitation	−0.07	−0.21, 0.08	4975
*US data, reptilian baseline (n = 227 measures from 15 species)*	(intercept)	−0.30	−4.95, 3.93	4591
	mass	0.14	−0.29, 0.66	4975
	sex (male)	−0.15	−0.46, 0.15	4975
	life history stage (non-breeding)	−0.92	−1.99, 0.08	5138
	max breeding attempts	−0.05	−0.89, 0.77	4975
	*life history stage* (non-breeding)^*^max breeding attempts	0.37	0.05, 0.67	5326
	temperature	0.04	−0.36, 0.41	4975
	*precipitation*	0.17	0.003, 0.34	5326

^*^Italicized text indicates that 95% CI did not overlap zero

## Results

### Avian corticosterone

**Figure 2:**
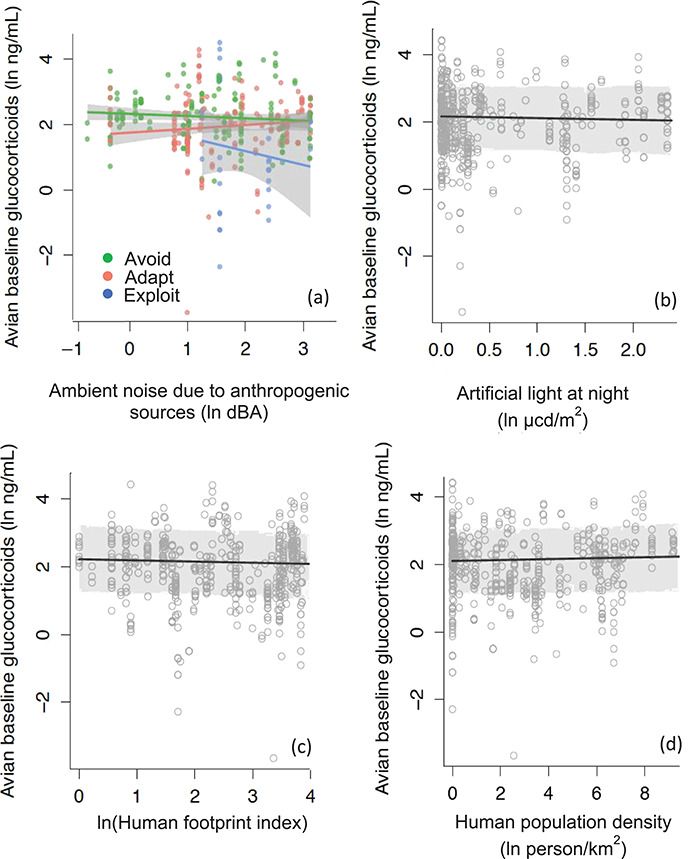
Relationships between (a) anthropogenic noise (data split by urban adaptability, US-only data), (b) ALAN (global data), (c) human footprint index (global data) and (d) human population density (global data) and avian baseline cort. Circles represent raw data points, whereas lines represent model outputs with 95% CI (shaded region). Figures were created by allowing the dependent variable to vary, while all other predictors were held constant.

In the restricted analysis of anthropogenic noise levels (which only included samples from the USA), the model that was ranked best fit included an interaction effect between anthropogenic noise levels and urban adaptability (Fig. 2a). However, other (non-intercept only) models were similarly ranked (ΔDIC < 1; Table 2). There was a negative relationship between baseline cort and anthropogenic noise levels for urban avoiders (β_avoider*noise_ = −0.36; 95% CI = −0.68, −0.03; Table 4), and, to a lesser extent, urban exploiters (β_exploiter*noise_ = −0.13; 95% CI = −0.83, 0.56; Table 4; Fig. 2a). For our global analysis of avian baseline cort, the model that included human population density, human footprint index and ALAN was ranked best fit; however, it had a ΔDIC < 1, compared to the other (non-intercept only) models (Table 2; Fig. 2b–d). Within this model, the parameter estimates of disturbance metrics were relatively small (β_human population density_ = 0.08; β_human footprint index_ = −0.05; β_ALAN_ = −0.24), and the 95% CIs overlapped zero for all three predictors (Table 4). Avian baseline cort varied over life history stage in both analyses, with baseline cort levels being lower in the non-breeding season, compared to the breeding season (*global model*; ß_non-breeding_ = −0.82, *US model*; ß _non-breeding_ = −0.87; Table 4). Additionally, temperature was negatively related to avian baseline cort in the global analysis (β_temperature_ = −0.28; 95% CI = −0.49, −0.06; Table 4).

We also did not find evidence for a general association between human footprint index, ALAN, human population density or anthropogenic noise and avian stress-induced cort, as these parameters were not in the best-fit models (Table 2). The urban adaptability parameter was in both best-fit models of stress-induced cort (global and US-only data); however, the 95% CIs overlapped zero in both models (Table 4). Our intercept-only models received <0.1% of the model weight in each analysis (Table 2).

### Reptilian corticosterone

For our analyses of baseline cort in reptiles, null models were the best fit; anthropogenic parameters did not improve model estimates (Table 3). Models that included human footprint index, human population density and ALAN received a similar amount of model weight as the null (14–26%; Table 3). The model that included anthropogenic noise, however, received considerably less weight than the null model (*null model*: 71%, *noise model*: 20%; Table 3). The 95% CIs overlapped zero for all contextual variables (temperature, precipitation, life history stage) in the top models of reptilian baseline cort (Table 4). However, the 95% CI for the interaction between life history stage and the maximum lifetime breeding attempts did not overlap zero (Table 4).

## Discussion

We found no general patterns in the association between human-induced environmental change and baseline or stress-induced cort in birds or baseline cort in reptiles. Our results only showed one context-specific pattern with regards to human-induced environmental change; for birds characterized as urban avoiders, baseline cort was negatively related to anthropogenic noise exposure (Fig. 2a). Therefore, our results partially supported our prediction that anthropogenic noise is negatively related to cort levels, but we found no general relationship between noise disturbance and cort across taxa in other contexts (Table 2). It is difficult to determine the reason why baseline cort was positively associated with anthropogenic noise for urban avoiders because of the correlative nature of this study. For example, exposure to chronic anthropogenic noise may cause urban avoiders to have lower baseline cort and indicate increased sensitivity to disturbance (as is found in some passerines experimentally exposed to anthropogenic noise; Table 1), or these species may be coping well with the stressor, indicating they are less sensitive to disturbance compared to urban adapter or exploiters. Interpreting increased or decreased baseline and stress-induced cort is also difficult due to context dependency (i.e. an individual or population’s previous exposure to disturbance), and interpretations tend to vary between physiologists, ecologists and conservationists.

Although the top-ranked model for avian baseline cort included ALAN (in addition to human footprint index and human population density), the negative relationship was relatively weak and opposite our hypothesis of a positive relationship between avian baseline cort and ALAN (Fig. 2b–d). We found no support for general associations between ALAN and stress-induced cort across birds and baseline cort across reptiles (Table 2). It is important to note that our results do not suggest that human-induced environmental change is not associated with changes in baseline and stress-induced cort within given bird and reptile populations, but rather that there is no general relationship between disturbance and cort across the collective data analyzed here.

This lack of a general pattern matches previous studies in these taxa (Table 1), as well as other recent comparative work on the effects of chronic stressors (both anthropogenic and non-anthropogenic) on animal physiology (Dickens and Romero, 2013; Tablado and Jenni, 2017), and may be explained by several non-mutually exclusive pathways. First, the physiological effects of chronic stress can differ among taxa (Dickens and Romero, 2013). For example, ALAN may extend foraging time for species that use visual, as opposed to acoustic, foraging cues, thus altering baseline and stress-induced cort through differences in nutritional state (Longcore and Rich, 2004). Second, the presence of other (non-anthropogenic) stressors, such as internal condition (Bonier *et al.*, 2009; Angelier *et al*., 2010), high predation risk (Clinchy *et al.*, 2011) or parasite load (Raouf *et al.*, 2006), may simultaneously impose varying selection pressures on cort and differ between taxa, and even individuals. These concurrent effects could mask our ability to detect general relationships between baseline and stress-induced cort and an individual, anthropogenic factor. This idea is supported by the fact that context, as determined by life history stage, was an important predictor in our models of avian baseline cort (Table 4). Third, the direction of change in baseline and/or stress-induced cort may differ based on an individual’s previous exposure (Grace and Anderson, 2018) and/or the length of exposure to a given stressor (i.e. habituation). Like many observational field studies, one limitation of our study is that we do not have information on a given individual’s previous exposure levels to anthropogenic disturbance (within a single breeding season or over one’s lifetime; French *et al*., 2008; Fokidis *et al.,* 2009; Foltz *et al*., 2015). Therefore, the degree to which our results may be influenced by previous habituation or adaptation to human-induced environmental change at the individual or population levels remains unknown. It is also important to acknowledge that our sample sizes may have been insufficient to detect any patterns of anthropogenic disturbance on baseline or stress-induced cort, due to the complexity of sampling free-living populations across different life history stages, environments etc. However, HormoneBase provides the most comprehensive database of cort levels currently available. Additionally, if sensitive species have already been lost in areas exposed to relatively high levels of human-induced environmental change, we may expect to find no such patterns between human induced environmental change and cort within the remaining species (i.e. those analyzed here).

Circulating baseline and stress-induced cort levels are often used to gauge the effect of human-induced environmental change on birds and reptiles (Lennox and Cooke, 2014); ~45% of publications in ‘*Conservation Physiology*’ over the past 5 years have focused on stress physiology (Madliger *et al.*, 2018). However, outside of our modest finding of a negative relationship between anthropogenic noise and baseline cort in birds characterized as urban avoiders, our results suggest that there is no general relationship between human-induced environmental change and baseline and stress-induced cort levels, as measured through plasma, in birds and reptiles. Therefore, directional predictions, such as predicting that bird and/or reptile populations exposed to high levels of human population density, human footprint index, anthropogenic noise or ALAN will have increased baseline or decreased stress-induced cort, should be made with caution; our data suggest that it is not possible to generalize the effects of human-induced environmental change on cort across species.

Research in the field of conservation physiology is highly valuable given increasing human-induced environmental change and our need to understand impacts on wildlife (Wikelski and Cooke, 2006; Madliger *et al.*, 2016). However, it is unlikely that any single physiological metric will accurately diagnose disturbed populations across species and contexts, as has been addressed in other reviews (Bonier *et al.*, 2009; Dickens and Romero, 2013; Sepp *et al.*, 2018; MacDougall-Shackleton *et al.*, 2019). Moving forward, measures of alternative physiological measures (e.g. changes in body mass, heart rate, oxidative stress, telomere length), or changes in glucocorticoid concentrations, alongside measures of behaviour (e.g. activity patterns, parental behaviour), population health (e.g. population size, birth rate, death rate) and survival may provide useful context to better understand potential negative relationships between human-induced environmental change and individual and population health.

## Supplementary Material

Supplement_coz110

## References

[ref1] Addis EA, Davis JE, Miner BE, Wingfield JC (2011) Variation in circulating corticosterone levels is associated with altitudinal range expansion in a passerine bird. Oecologia 167: 369–378. 21533815 10.1007/s00442-011-2001-5

[ref2] Alaasam VJ, Duncan R, Casagrande S, Davies S, Sidher A, Seymoure B, Shen Y, Zhang Y, Ouyang JQ (2018) Light at night disrupts nocturnal rest and elevates glucocorticoids at cool color temperatures. J Exp Zool A 329: 465–472. 10.1002/jez.2168 PMC6205889 29766666

[ref3] Angelier F, Wingfield JC, Weimerskirch H, Chastel O (2010) Hormonal correlates of individual quality in a long-lived bird: a test of the ‘corticosterone–fitness hypothesis’. Biol Lett 6: 846. 20573614 10.1098/rsbl.2010.0376 PMC3001372

[ref4] Atwell JW, Cardoso GC, Whittaker DJ, Campbell-Nelson S, Robertson KW, Ketterson ED (2012) Boldness behavior and stress physiology in a novel urban environment suggest rapid correlated evolutionary adaptation. Behav Ecol 23: 960–969. 22936840 10.1093/beheco/ars059 PMC3431113

[ref5] Baxter-Gilbert JH, Riley JL, Mastromonaco GF, Litzgus JD, Lesbarrères D (2014) A novel technique to measure chronic levels of corticosterone in turtles living around a major roadway. Conserv Physiol 2: cou036. 27293657 10.1093/conphys/cou036 PMC4806746

[ref6] Benítez-López A, Alkemade R, Verweij PA (2010) The impacts of roads and other infrastructure on mammal and bird populations: a meta-analysis. Biol Conserv 143: 1307–1316.

[ref7] Bennie J, Davies TW, Duffy JP, Inger R, Gaston KJ (2014) Contrasting trends in light pollution across europe based on satellite observed night time lights. Sci Rep 4: 3789. 24445659 10.1038/srep03789 PMC3896907

[ref8] Blair RB (2001) Birds and butterflies along urban gradients in two ecoregions of the United States: is urbanization creating a homogeneous fauna In Biotic Homogenization. Springer, Boston, MA, pp. 33–56.

[ref9] Bolker BM, Brooks ME, Clark CJ, Geange SW, Poulsen JR, Stevens MHH, White JSS (2009) Generalized linear mixed models: a practical guide for ecology and evolution. Trends Ecol Evol 24: 127–135. 19185386 10.1016/j.tree.2008.10.008

[ref10] Bonier F (2012) Hormones in the city: endocrine ecology of urban birds. Horm Behav 61: 763–772. 22507448 10.1016/j.yhbeh.2012.03.016

[ref11] Bonier F, Martin PR, Moore IT, Wingfield JC (2009) Do baseline glucocorticoids predict fitness. Trends Ecol Evol 24: 634–642. 19679371 10.1016/j.tree.2009.04.013

[ref12] Breiman L (2001) Random forests. Mach Learn 45: 5–32.

[ref13] Busch DS, Hayward LS (2009) Stress in a conservation context: a discussion of glucocorticoid actions and how levels change with conservation-relevant variables. Biol Conserv 142: 2844–2853.

[ref14] Buxton RT, McKenna MF, Mennitt D, Fristrup K, Crooks K, Angeloni L, Wittemyer G (2017) Noise pollution is pervasive in us protected areas. Science 356: 531–533. 28473587 10.1126/science.aah4783

[ref15] Center for International Earth Science Information Network (CIESIN), Columbia University (2016) Gridded population of the world, version 4 (gpwv4): population density. NASA Socioeconomic Data and Applications Center (SEDAC), Palisades, NY.

[ref16] Clinchy M, Zanette L, Charlier TD, Newman AEM, Schmidt KL, Boonstra R, Soma KK (2011) Multiple measures elucidate glucocorticoid responses to environmental variation in predation threat. Oecologia 166: 607–614. 21279653 10.1007/s00442-011-1915-2

[ref17] Davies S, Haddad N, Ouyang JQ (2017) Stressful city sounds: glucocorticoid responses to experimental traffic noise are environmentally dependent. Biol Lett 13: 20170276. 29046372 10.1098/rsbl.2017.0276 PMC5665767

[ref18] Dickens MJ, Romero LM (2013) A consensus endocrine profile for chronically stressed wild animals does not exist. Gen Comp Endocrinol 191: 177–189. 23816765 10.1016/j.ygcen.2013.06.014

[ref19] Falchi F, Cinzano P, Duriscoe D, Kyba CC, Elvidge CD, Baugh K, Portnov BA, Rybnikova NA, Furgoni R (2016a) The new world atlas of artificial night sky brightness. Sci Adv 2: e1600377. 27386582 10.1126/sciadv.1600377 PMC4928945

[ref20] Falchi F, Cinzano P, Duriscoe D, Kyba CCM, Elvidge CD, Baugh K, Portnov B, Rybnikova NA, Furgoni R (2016b). Supplement to: The new world atlas of artificial night sky brightness. GFZ Data Services. doi:10.5880/GFZ.1.4.2016.001 PMC4928945 27386582

[ref21] Fokidis HB, Orchinik M, Deviche P (2009) Corticosterone and corticosteroid binding globulin in birds: relation to urbanization in a desert city. Gen Comp Endocrinol 160: 259–270. 19116155 10.1016/j.ygcen.2008.12.005

[ref22] Fokidis HB, Orchinik M, Deviche P (2011) Context-specific territorial behavior in urban birds: no evidence for involvement of testosterone or corticosterone. Horm Behav 59: 133–143. 21078324 10.1016/j.yhbeh.2010.11.002

[ref23] Foltz SL, Davis JE, Battle KE, Greene VW, Laing BT, Rock RP, Ross AE, Tallant JA, Vega RC, Moore IT (2015) Across time and space: effects of urbanization on corticosterone and body condition vary over multiple years in song sparrows (*Melospiza melodia*). J Exp Zool A Ecol Genet Physiol 323: 109–120. 25678475 10.1002/jez.1906

[ref24] French SS, Fokidis HB, Moore MC (2008) Variation in stress and innate immunity in the tree lizard (*Urosaurus ornatus*) across an urban–rural gradient. J Comp Physiol B 178: 997–1005. 18594834 10.1007/s00360-008-0290-8 PMC2774757

[ref25] French SS, Neuman-Lee LA, Terletzky PA, Kiriazis NM, Taylor EN, DeNardo DF (2017) Too much of a good thing? Human disturbance linked to ecotourism has a “dose-dependent” impact on innate immunity and oxidative stress in marine iguanas, *Amblyrhynchus cristatus*. Biol Conserv 210: 37–47.

[ref26] French SS, Webb AC, Hudson SB, Virgin EE (2018) Town and country reptiles: a review of reptilian responses to urbanization. Integr Comp Biol 58: 948–966. 29873730 10.1093/icb/icy052

[ref27] Goolsby EW, Bruggeman J, Ané C (2016) Rphylopars: fast multivariate phylogenetic comparative methods for missing data and within-species variation. Methods Ecol Evol 8: 22–27.

[ref28] Grace JK, Anderson DJ (2018) Early-life maltreatment predicts adult stress response in a long-lived wild bird. Biol Lett 14: 20170679. 29321248 10.1098/rsbl.2017.0679 PMC5803595

[ref29] Grunst ML, Rotenberry JT, Grunst AS (2014) Variation in adrenocortical stress physiology and condition metrics within a heterogeneous urban environment in the song sparrow *Melospiza melodia*. J Avian Biol 45: 574–583.

[ref30] Hadfield JD (2010) MCMC methods for multi-response generalized linear mixed models: the MCMCglmm r package. J Stat Softw 33: 1–22.20808728

[ref31] Harris I, Jones PD, Osborn TJ, Lister DH (2014) Updated high-resolution grids of monthly climatic observations–the cru ts3. 10 dataset. Int J Climatol 34: 623–642.

[ref32] Injaian AS, Taff CC, Pearson KL, Gin MMY, Patricelli GL, Vitousek MN (2018) Effects of experimental chronic traffic noise exposure on adult and nestling corticosterone levels, and nestling body condition in a free-living bird. Horm Behav 106: 19–27. 30189211 10.1016/j.yhbeh.2018.07.012

[ref33] Janin A, Léna J-P, Joly P (2011) Beyond occurrence: body condition and stress hormone as integrative indicators of habitat availability and fragmentation in the common toad. Biol Conserv 144: 1008–1016.

[ref34] Johnson MA, Francis CD, Miller ET, Downs CJ, Vitousek MN (2018) Detecting bias in large-scale comparative analyses: methods for expanding the scope of hypothesis-testing with hormonebase. Integr Comp Biol 58: 720–728. 29873731 10.1093/icb/icy045

[ref35] Kleist NJ, Guralnick RP, Cruz A, Lowry CA, Francis CD (2018) Chronic anthropogenic noise disrupts glucocorticoid signaling and has multiple effects on fitness in an avian community. Proc Natl Acad Sci 201709200. 10.1073/pnas.1709200115 PMC5789909 29311304

[ref36] Krause JS, Dorsa D, Wingfield JC (2014) Changes in plasma concentrations of progesterone, dehydroepiandrosterone and corticosterone in response to acute stress of capture, handling and restraint in two subspecies of white-crowned sparrows. Comp Biochem Physiol A 177: 35–40. 10.1016/j.cbpa.2014.07.019 25072921

[ref37] Kumar S, Stecher G, Suleski M, Hedges SB (2017) Timetree: a resource for timelines, timetrees, and divergence times. Mol Biol Evol 34: 1812–1819. 28387841 10.1093/molbev/msx116

[ref38] Kvamme BO, Gadan K, Finne-Fridell F, Niklasson L, Sundh H, Sundell K, Taranger GL, Evensen O (2013) Modulation of innate immune responses in the Atlantic salmon by chronic hypoxia-induced stress. Fish Shellfish Immun 34: 55–65. 10.1016/j.fsi.2012.10.006 23085636

[ref39] Lennox R, Cooke SJ (2014) State of the interface between conservation and physiology: a bibliometric analysis. Conserv Physiol 2: cou003. 27293624 10.1093/conphys/cou003 PMC4732491

[ref40] Longcore T, Rich C (2004) Ecological light pollution. Front Ecol Environ 2: 191–198.

[ref41] Lucas LD, French SS (2012) Stress-induced tradeoffs in a free-living lizard across a variable landscape: consequences for individuals and populations. PLoS One 7: e49895. 23185478 10.1371/journal.pone.0049895 PMC3502225

[ref42] MacDougall-Shackleton SA, Bonier F, Romero LM, Moore IT (2019) Glucocorticoids and “stress” are not synonymous. Int Org Biol 1: obz017. 10.1093/iob/obz017 PMC7671118 33791532

[ref43] Madliger CL, Cooke SJ, Crespi EJ, Funk JL, Hultine KR, Hunt KE, Rohr JR, Sinclair BJ, Suski CD, Willis CK (2016) Success stories and emerging themes in conservation physiology. Conserv Physiol 4: cov057. 10.1093/conphys/cov057 PMC4922248 27382466

[ref44] Madliger CL, Love OP (2015). The power of physiology in changing landscapes: considerations for the continues integrations of conservation and physiology. Int Comp Bio 55: 545–553. 10.1093/icb/icv001 25805172

[ref45] Madliger CL, Love OP, Hultine KR, Cooke SJ (2018) The conservation physiology toolbox: status and opportunities. Conserv Physiol 6: coy029. 10.1093/conphys/coy029 PMC6007632 29942517

[ref46] Martin LB et al. (2018) IUCN conservation status does not predict glucocorticoid concentrations in reptiles and birds. Integr Comp Biol 58: 800–813. 30052988 10.1093/icb/icy102

[ref47] Monaghan P, Haussmann MF (2015) The positive and negative consequences of stressors during early life. Early Hum Dev 91: 643–647. 26385447 10.1016/j.earlhumdev.2015.08.008 PMC4706554

[ref48] NPS (2014) Geospatial sound modeling. Natural Sounds and Night Skies and Inventory and Monitoring Divisions https://irma.nps.gov/DataStore/Reference/Profile/2217356.

[ref49] Ouyang JQ, Isaksson C, Schmidt C, Hutton P, Bonier F, Dominoni D (2018) A new framework for urban ecology: an integration of proximate and ultimate responses to anthropogenic change. Int Comp Biol 58: 915–928. 10.1093/icb/icy110 PMC6204990 30376106

[ref50] Ouyang JQ, de Jong M, Hau M, Visser ME, van Grunsven RH, Spoelstra K (2015) Stressful colours: corticosterone concentrations in a free-living songbird vary with the spectral composition of experimental illumination. Biol Lett 11: 20150517. 26311159 10.1098/rsbl.2015.0517 PMC4571683

[ref51] Owen DA, Carter ET, Holding ML, Islam K, Moore IT (2014) Roads are associated with a blunted stress response in a North American pit viper. Gen Comp Endocrinol 202: 87–92. 24798578 10.1016/j.ygcen.2014.04.020

[ref52] Partecke J, Schwabl I, Gwinner E (2006) Stress and the city: urbanization and its effects on the stress physiology in European blackbirds. Ecology 87: 1945–1952. 16937632 10.1890/0012-9658(2006)87[1945:satcua]2.0.co;2

[ref53] Polich RL (2016) Stress hormone levels in a freshwater turtle from sites differing in human activity. Conserv Physiol 4: cow016. 10.1093/conphys/cow016 PMC4892809 27293763

[ref54] Potvin DA, MacDougall-Shackleton SA (2015) Traffic noise affects embryo mortality and nestling growth rates in captive zebra finches. J Exp Zool A Ecol Genet Physiol 323: 722–730. 26349453 10.1002/jez.1965

[ref55] Raouf SA, Smith LC, Brown MB, Wingfield JC, Brown CR (2006) Glucocorticoid hormone levels increase with group size and parasite load in cliff swallows. Anim Behav 71: 39–48.

[ref56] Rich EL, Romero LM (2005) Exposure to chronic stress downregulates corticosterone responses to acute stressors. Am J Physiol Regul Integr Comp Physiol 288: R1628–R1636. 15886358 10.1152/ajpregu.00484.2004

[ref57] Riitters KH, Wickham JD (2003) How far to the nearest road. Front Ecol Environ 1: 125–129.

[ref59] Russ A, Reitemeier S, Weissmann A, Gottschalk J, Einspanier A, Klenke R (2015) Seasonal and urban effects on the endocrinology of a wild passerine. Ecol Evol 5: 5698–5710. 27069618 10.1002/ece3.1820 PMC4813110

[ref60] Sepp T, McGraw KJ, Kaasik A, Giraudeau M (2018) A review of urban impacts on avian life-history evolution: does city living lead to slower pace of life. Global Change Biol 24: 1452–1469. 10.1111/gcb.13969 29168281

[ref61] Strasser EH, Heath JA (2013) Reproductive failure of a human-tolerant species, the American kestrel, is associated with stress and human disturbance. J Appl Ecol 50: 912–919.

[ref62] Swaddle JP et al. (2015) A framework to assess evolutionary responses to anthropogenic light and sound. Trends Ecol Evol 30: 550–560. 26169593 10.1016/j.tree.2015.06.009

[ref63] Tablado Z, Jenni L (2017) Determinants of uncertainty in wildlife responses to human disturbance. Biol Rev 92: 216–233. 26467755 10.1111/brv.12224

[ref64] Tarlow EM, Blumstein DT (2007) Evaluating methods to quantify anthropogenic stressors on wild animals. Appl Anim Behav Sci 102: 429–451.

[ref65] Thaker M, Lima SL, Hews DK (2009) Acute corticosterone elevation enhances antipredator behaviors in male tree lizard morphs. Horm Behav 56: 51–57. 19281811 10.1016/j.yhbeh.2009.02.009

[ref66] Venter O, Sanderson EW, Magrach A, Allan JR, Beher J, Jones KR, Possingham HP, Laurance WF, Wood P, Fekete BM (2016a) Global terrestrial human footprint maps for 1993 and 2009. Scientific Data 3: 160067. 27552448 10.1038/sdata.2016.67 PMC5127486

[ref67] Venter O, Sanderson EW, Magrach A, Allan JR, Beher J, Jones KR, Possingham HP, Laurance WF, Wood P, Fekete BM (2016b) Sixteen years of change in the global terrestrial human footprint and implications for biodiversity conservation. Nat Comm 7: 12558. 10.1038/ncomms12558 PMC4996975 27552116

[ref68] Vitousek MN et al. (2018) Hormonebase, a population-level database of steroid hormone levels across vertebrates. Sci Data 5: 180097. 10.1038/sdata.2018.97 PMC5963335 29786693

[ref69] Vitousek MN et al. (2019) Macroevolutionary patterning in glucocorticoids suggests different selective pressures shape baseline and stress-induced levels. Am Nat 193: 866–880. 31094598 10.1086/703112

[ref70] Weaver M, Gao S, McGraw KJ (2018) Circulating corticosterone levels vary during exposure to anthropogenic stimuli and show weak correlation with behavior across an urban gradient in house finches (*Haemorhous mexicanus*). Gen Comp Endocrinol 266: 52–59. 29673843 10.1016/j.ygcen.2018.04.017

[ref71] Wikelski M, Cooke SJ (2006) Conservation physiology. Trends Ecol Evol 21: 38–46. 16701468 10.1016/j.tree.2005.10.018

[ref72] Wingfield JC, Kitaysky AS (2002) Endocrine responses to unpredictable environmental events: stress or anti-stress hormones. Integr Comp Biol 42: 600–609. 21708756 10.1093/icb/42.3.600

[ref73] Zhang S, Lei F, Liu S, Li D, Chen C, Wang P (2011) Variation in baseline corticosterone levels of tree sparrow (*Passer montanus*) populations along an urban gradient in Beijing, China. J Ornithol 152: 801–806.

