# Supplementary material for: Baseline and stress-induced corticosterone levels across birds and reptiles do not reflect urbanization levels
Source: Conserv Physiol. 2020 Jan 27;8(1):coz110. doi: 10.1093/conphys/coz110 (PMC6978728; doi:10.1093/conphys/coz110)
Supplement: Supplement_coz110 [file supplement_coz110.docx]

| **Table S1**. Bird species included in this analysis, characterized by the urban adaptability (adapter, avoider, exploiter). Identifying information for the paper used to characterize each species is provided (DOI or Stable link), as well as the justification (Notes). If not enough information was present in the literature to categorize a species’ urban adaptability, they were given a ‘1’ in the Insufficient Data column and not included in the analysis. | | | | | |
| --- | --- | --- | --- | --- | --- |
| **SpeciesName** | **Urban**  **Adaptability** | **Insufficient Data** | **DOI** | **StableLink** | **Notes** |
| Accipiter_cooperii | Adapt | 0 | 10.1650/0010-5422(2003)105[107:FBOCHA]2.0.CO;2 |  | Readily nest in both urban and suburban areas, with dense populations in urban areas perhaps due to high prey availability |
| Accipiter_gentilis | Adapt | 0 | 10.1111/j.1365-2656.2008.01420.x |  | Have colonized cities over the past few decades, with number in urban areas now being equal or greater than in the rural periphery |
| Accipiter_striatus | Adapt | 0 | 10.3356/JRR-13-32.1 |  | Present in areas with > 50% urban cover, but their abundance is greater abundance in areas with lower urban cover (>30%) |
| Agelaius_phoeniceus | Adapt | 0 | 10.1002/ecs2.2517 |  | Present in urban landscapes (~ 2-3 birds/ha, but densities are greater in grassland patches (~4-5 birds/ha) |
|  |  |  |  |  |  |
| Anas_acuta | Adapt | 1 | 10.1016/j.landurbplan.2011.04.007 |  | Present in urban parks, but only during non-breeding period (breeds in freshwater marshes, small lakes, and rivers with dense vegetation cover) |
| Aphelocoma_coerulescens | Adapt | 0 | NA | https://search.proquest.com/docview/211267454?accountid=10267 | Common in suburban and wildland habitat |
| Bombycilla_cedrorum | Adapt | 0 | 10.1016/j.biocon.2005.09.041 |  | Present in urban habitats, positively associated with urbanization |
| Calidris_melanotos | Adapt | 1 | 10.1016/j.landurbplan.2011.04.007 |  | Present in urban parks, but only during non-breeding period (breeds in Arctic tundra) |
| Calidris_pusilla | Adapt | 1 | 10.1016/j.landurbplan.2011.04.007 |  | Present in urban parks, but only during non-breeding period (breeds in Arctic wet sedge and tundra) |
| Cantorchilus_leucotis | Adapt | 1 | 10.1016/j.envpol.2018.01.045 |  | Present in forest fragments close to urban area/airport |
| Cardinalis_cardinalis | Adapt | 0 | 10.1890/11-0022.1 |  | Common across an urban:rural gradient |
| Catharus_guttatus | Adapt | 1 | 10.1016/j.landurbplan.2007.06.010 |  | Present in urban parks and golf courses, but at very lower numbers (2%). Mainly breeds in forest habitats with dense canopy cover. |
| Catharus_ustulatus | Adapt | 1 | 10.1525/cond.2010.090049 |  | Use stopover sites equally, despite associated levels of urbanization |
| Dumetella_carolinensis | Adapt | 0 | 10.1016/j.landurbplan.2010.11.015 |  | Present at both urban and rural sites, but presence at urban sites is slightly greater |
| Empidonax_oberholseri | Adapt | 0 | 10.1007/978-1-4615-1531-9_3 |  | Clear-cut areas associated with increased abundance. Also, present in urban parks in Mexico City, with > 80% built cover within 1 km of the park |
| Empidonax_occidentalis | Adapt | 0 | 10.1007/978-1-4615-1531-9_3 |  | Abundances don't decrease as a result of partial cut of forest. Also, present in urban parks in Mexico City, with > 80% built cover within 1 km of the park |
| Fringilla_coelebs | Adapt | 0 | 10.1525/cond.2008.8409 |  | Present in some urban areas, but more common at rural sites |
| Haemorhous_mexicanus | Adapt | 0 | 10.1139/z79-307 |  | Present in urban areas, but often avoid commercial and industrial habitats |
| Hirundo_rustica | Adapt | 0 | 10.1525/cond.2008.8409 |  | Commonly present across an urban:rural gradient, and in urban parks |
| Junco_hyemalis | Adapt | 0 | 10.1016/j.tree.2005.11.019 |  | Generally considered avoiders, but one well documented population colonized San Diego, CA, USA |
| Melospiza_melodia | Adapt | 0 | 10.1016/j.biocon.2005.09.041 |  | Present in urbanized areas, but abundance is negatively associated with urbanization |
| Melozone_aberti | Adapt | 0 | 10.1111/j.2008.0908-8857.04248.x |  | Inhabits only desert riparian regions of the southwestern US, but common in Phoenix, AZ, USA |
| Mimus_polyglottos | Adapt | 0 | 10.2307/2269387 |  | Maximum densities reached at a residential area |
| Molothrus_aeneus | Adapt | 0 | 10.2307/1368320 |  | Common in urban areas with both exotic and urban plant species |
| Molothrus_ater | Adapt | 0 | 10.2307/1368320 |  | Common in urban areas with both exotic and urban plant species |
| Parus_atricapillus | Adapt | 0 | 10.1650/0010-5422(2005)107[0678:EOUSOS]2.0.CO;2 |  | Highest abundance in suburban areas, abundance in urban areas lower than in natural habitats |
| Parus_caeruleus | Adapt | 0 | 10.1525/cond.2008.8409 |  | Highest in areas that have tree coverage with bare ground underneath (habitat that could be found along an urban:rural gradient) |
| Parus_major | Adapt | 0 | 10.1525/cond.2008.8409 |  | Highest in areas that have tree coverage with bare ground underneath (habitat that could be found along an urban:rural gradient) |
| Parus_rufescens | Adapt | 0 | 10.2307/1367699 |  | Present in urban parks, but numbers decrease as modification of vegetation increased and park size decreased. |
| Phylloscopus_trochilus | Adapt | 1 | 10.1007/BF01640537 |  | Present in some urban areas, but infrequently enough that they have previously not been classified as an urban adapter. |
| Scardafella_inca | Adapt | 0 | 10.1016/S0169-2046(02)00096-8 |  | Common in golf courses in Phoenix, AZ, USA |
| Sialia_sialis | Adapt | 0 | 10.1007/s11252-012-0265-0 |  | Populations in the US have rebounded thanks to nest boxes in managed landscapes |
| Sitta_europaea | Adapt | 0 | 10.1023/A:1011190902041 |  | Resident of mature broadleaves woodland, but nests is urban parks (in this study, they were only missing in the smallest, most isolated patches in the urban environment) |
| Spinus_tristis | Adapt | 0 | 10.1007/978-3-319-43314-1_2 |  | Present in both urban and intact vegetation, almost equally |
| Spizella_arborea | Adapt | 1 | 10.1016/j.landurbplan.2011.04.007 |  | Observed in urban parks in St. Louis, MO, USA during overwintering and common at feeders, but breeds in the artic-subarctic transition zone. |
| Sturnus_vulgaris | Adapt | 0 | 10.1016/j.biocon.2005.09.041 |  | Abundance is positively associated with urbanization |
| Sylvia_atricapilla | Adapt | 1 | 10.1016/j.landurbplan.2008.03.006 |  | Associated with medium-low density of buildings in one study, but described as avoiding urban areas in another |
| Sylvia_curruca | Adapt | 0 | 10.1023/A:1009505418327 |  | Present in urban parks, but not if the park was < 0.75 ha |
| Tachycineta_bicolor | Adapt | 0 | 10.1016/0304-4009(86)90012-4 |  | Nest in riparian areas, and are common in suburban areas |
| Toxostoma_curvirostre | Adapt | 0 | 10.1016/S0169-2046(02)00096-8 |  | Present throughout Phoenix, AZ, USA |
| Troglodytes_aedon | Adapt | 0 | 10.1016/j.biocon.2005.09.041 |  | Modestly positively associated with urbanization |
| Turdus_grayi | Adapt | 0 | 10.1111/j.1474-919X.2008.00899.x |  | Present in suburban/urban areas, but have higher reproductive success in more rural areas |
| Turdus_migratorius | Adapt | 0 | 10.1525/auk.2009.07136 |  | Have greater abundances in suburban areas, compared to forested areas |
| Tyto_alba | Adapt | 0 | 10.1676/11-173.1 |  | Breed in both urban and rural areas, but more common in rural |
| Zenaida_asiatica | Adapt | 0 | 10.2307/1366729 |  | Common in urban areas of Tucson, AZ, USA |
| Zonotrichia_albicollis | Adapt | 0 | 10.3312/jyio1952.21.178 |  | Breed in the edge habitat in boreal forests in Northeastern US and Canada, but listed as having high density in urban habitats and has been found to collide with buildings in urban areas (suggesting present) |
| Zonotrichia_leucophrys | Adapt | 0 | 10.1093/beheco/arl050 |  | Breed in both urban (San Francisco, CA, USA and Seattle, WA, USA and rural sites) |
| Aethia_cristatella | Avoid | 0 | 10.1002/jwmg.21175 |  | Breed on islands in Alaska, USA |
| Aix_sponsa | Avoid | 0 | 10.1016/j.landurbplan.2003.08.009 |  | Breed in forested and woodland habitats near or next to creeks, sloughs, ponds and streams |
| Alle_alle | Avoid | 0 | 10.14430/arctic2526 |  | Breed in talus in Greenland |
| Amphispiza_bilineata | Avoid | 0 | 10.1890/1051-0761(2003)013[0530:LSPOBT]2.0.CO;2 |  | Breed in desert habitats, with core of its range being in the northern Chihuahua Desert (south-central New Mexico, USA) |
| Calcarius_lapponicus | Avoid | 0 | NA | https://www.jstor.org/stable/4085407 | Breed in the Arctic |
| Calcarius_mccownii | Avoid | 0 | NA | https://www.fs.usda.gov/treesearch/pubs/32123 | Breed in grassland prairies |
| Calcarius_ornatus | Avoid | 0 | NA | https://www.fs.usda.gov/treesearch/pubs/32123 | Breed in grassland prairies |
| Calcarius_pictus | Avoid | 0 | NA | https://www.fs.usda.gov/treesearch/pubs/32123 | Breed in grassland prairies |
| Calidris_mauri | Avoid | 0 | NA | https://www.researchgate.net/profile/David_Lank/publication/228610976_Conservation_Plan_for_the_Western_Sandpiper_Calidris_mauri/links/0fcfd505c6fe666052000000.pdf | Breed in the Arctic, and human developing to wintering grounds (coastal wetland) have had negative impacts on sandpepr conservation |
| Callipepla_gambelii | Avoid | 0 | 10.1016/S0140-1963(18)31402-2 |  | Breed in the desert of southwestern North America, distribution is limited by suitable food resources and cover |
| Calonectris_diomedea | Avoid | 0 | 10.1080/00063650009461185 |  | Breed at high densities the sandy soil of coastal zone of the Azores only (very few burrows in inland habitats) |
| Campylorhynchus_brunneicapillus | Avoid | 0 | 10.1111/mec.13176 |  | Isolated to habitats with prickly pear cactus |
| Cardellina_rubrifrons | Avoid | 0 | 10.2307/3801907 |  | Less abundant in more modified habitats, nest in dense foliage |
| Carduelis_hornemanni | Avoid | 0 | 10.1016/j.ympev.2008.03.027 |  | Breed in the arctic |
| Centropus_grillii | Avoid | 0 | 10.1016/j.anbehav.2003.12.012 |  | Breed in flooded African grasslands |
| Cettia_diphone | Avoid | 0 | 10.1023/A:1008869410668 |  | Decreasing occurrence in woodland areas of smaller size |
| Diomedea_exulans | Avoid | 0 | 10.1111/j.2006.0908-8857.03675.x |  | Nest on islands off the southern tips of Africa and Australia |
| Eudyptes_chrysocome | Avoid | 0 | 10.3354/meps240273 |  | Breed in the sub-Antarctic |
| Ficedula_hypoleuca | Avoid | 0 | 10.1016/j.landurbplan.2005.04.003 |  | Nest in mature forests, not present in urban areas |
| Fregata_minor | Avoid | 0 | 10.1016/0006-3207(83)90006-X |  | Breed on islands, negatively impacted by human presence |
| Fringilla_montifringilla | Avoid | 0 | 10.1016/S0169-2046(97)00089-3 |  | Presence negatively correlated with human population density, not present in areas with medium (200 - 499 people/km^2^) to high urbanization (> 500 people/km^2^) levels |
| Geothlypis_trichas | Avoid | 0 | 10.1890/04-0927 |  | Abundance is positively associated with canopy closure (prefer fields with more shrub cover) |
| Hemignathus_virens | Avoid | 0 | 10.1017/S0959270906000244 |  | Present in mid to high altitude wet forests, limited to these areas due to anthropogenic factors at lower altitudes |
| Himatione_sanguinea | Avoid | 0 | 10.1093/auk/95.3.518 |  | Breed in forests in Hawaii, USA |
| Histrionicus_histrionicus | Avoid | 0 | 10.1675/1524-4695(2002)025[0333:AADOHD]2.0.CO;2 |  | Breed on islands off the cost of Canada (near Newfoundland) |
| Hylocichla_mustelina | Avoid | 0 | 10.1016/j.biocon.2005.09.041 |  | Abundance is negatively associated with urbanization |
| Hylophylax_naevioides | Avoid | 0 | 10.1007/s00436-018-5955-y |  | Breed in tropical forests |
| Luscinia_svecica | Avoid | 1 | 10.1016/j.landurbplan.2013.10.005 |  | Ranges from artic to steppe habitats, one individual observed in 4% of urban gardens surveyed during migratory period |
| Macronectes_giganteus | Avoid | 0 | 10.1071/MU05033 |  | Breed on sub-Antarctic islands |
| Macronectes_halli | Avoid | 0 | 10.1007/s00300-006-0137-2 |  | Breed on sub-Antarctic islands and has increased heart rate when human visitors come within 40 m |
| Melozone_fuscus | Avoid | 0 | 10.2307/3671643 |  | Inhabit only desert regions of the southwestern US |
| Montifringilla_taczanowskii | Avoid | 0 | NA | http://citeseerx.ist.psu.edu/viewdoc/download?doi=10.1.1.452.5815&rep=rep1&type=pdf | Breed in pika burrows in alpine meadows |
| Oceanodroma_tethys | Avoid | 0 | 10.2307/1521313 |  | Breed in Galapagos Islands, off the coast of Chile |
| Oreothlypis_celata | Avoid | 1 | 10.1525/cond.2010.100062 |  | Breed across Canada/Alaska, Found in city (Morelia) during migration |
| Passerculus_sandwichensis | Avoid | 0 | 10.5751/ACE-00304-040201 |  | Salt marsh obligate that is negatively affected by pedestrian ecotourism |
| Peucaea_carpalis | Avoid | 0 | 10.1676/18-57 |  | An endemic bird of the arid coastal plains of Sonora, northern Sinaloa, and southern Arizona |
| Phalacrocorax_harrisi | Avoid | 0 | 10.7589/0090-3558-46.3.1005 |  | Flightless bird that breeds mainly (>95%) on Fernandina and Isabela Islands |
| Phalaropus_fulicarius | Avoid | 0 | 10.1111/j.1474-919X.1988.tb00974.x |  | Breed in the Arctic tundra |
| Picoides_borealis | Avoid | 0 | 10.1016/S0006-3207(98)00019-6 |  | Endangered bird endemic to mature pine forests in the southeastern US |
| Plectrophenax_nivalis | Avoid | 0 | 10.1007/s00300-014-1595-6 |  | Breed in the high Arctic region |
| Pterodroma_macroptera | Avoid | 0 | 10.1016/j.biocon.2013.11.002 |  | Breed on Northshore islands off the coast of New Zealand |
| Ptilotula_penicillatus | Avoid | 0 | 10.1016/j.biocon.2009.10.010 |  | Breed in eucalypt woodlands |
| Ramphocelus_dimidiatus | Avoid | 0 | 10.1086/285480 |  | Neotropical bird common in both young, scattered, second-growth forest and along edges of fairly mature forest in Panama |
| Rissa_tridactyla | Avoid | 0 | 10.1890/0012-9658(1998)079[2415:CRSABH]2.0.CO;2 |  | Cliff-nesting seabird |
| Setophaga_caerulescens | Avoid | 0 | 10.2307/5721 |  | Forest dwellers in Northern US hardwood forests |
| Setophaga_chrysoparia | Avoid | 0 | NA | https://www.cambridge.org/core/journals/animal-conservation-forum/article/winter-habitat-and-distribution-of-the-endangered-goldencheeked-warbler-dendroica-chrysoparia/CCDF789E07EBBA1D5C545B387A426D9C/core-reader | Endangered bird restricted to 18 Texas, USA counties, urban expansion thought to be a contributing factor to decline |
| Setophaga_coronata | Avoid | 1 | 10.1642/0004-8038(2004)121[0046:FAALDO]2.0.CO;2 |  | Broad range (Alaska to Guatemala), but detected most often in regeneration habitats and loblolly pine plots |
| Setophaga_petechia | Avoid | 0 | 10.1111/j.1365-2664.2011.01978.x |  | Restricted to riparian areas. |
| Somateria_mollissima | Avoid | 0 | 10.1007/s004420050238 |  | Circumpolar distribution |
| Somateria_spectabilis | Avoid | 0 | NA | https://www.jstor.org/stable/40512230 | Circumpolar distribution |
| Spheniscus_magellanicus | Avoid | 0 | 10.1093/auk/108.4.923 |  | Forage in ocean and breed (in burrows) on island and mainland coastal habitats along the southern tip of South America |
| Spheniscus_mendiculus | Avoid | 0 | 10.2989/02577618409504370 |  | Breed in burrows on islands off the coast of Africa, susceptible to human disturbance. Government built walls around many of the breeding islands to keep humans out. |
| Spinus_pinus | Avoid | 0 | 10.1016/j.ufug.2011.03.004 |  | Breed in open coniferous forests. Present only in reserve, not in urban parks |
| Spizella_pusilla | Avoid | 0 | 10.1890/04-0927 |  | More abundant in rural, as compared to urban sites |
| Sterna_hirundo | Avoid | 0 | 10.2307/1521499 |  | Island nesters that choose open colony sites with minimal vegetation |
| Sula_nebouxii | Avoid | 0 | 10.2307/1521995 |  | Galapagos island water bird that flees from its nest when disturbed by tourists |
| Synthliboramphus_antiquus | Avoid | 0 | 10.1139/z90-064 |  | Colonial, burrow-nesting alcid that nests on conifer-forested slopes within a few hundred meters of sea, usually on small islands |
| Thalassarche_melanophrys | Avoid | 0 | 10.1071/MU07028 |  | Circumpolar distribution |
| Uria_lomvia | Avoid | 0 | 10.1111/j.1365-2656.2005.00982.x |  | Breed only in the Arctic and Subarctic |
| Vestiaria_coccinea | Avoid | 0 | 10.3133/ofr20131150 |  | Restricted to high-elevation forests on Hawaii, Maui and Kauai, USA |
| Vireo_griseus | Avoid | 0 | 10.1007/978-1-4615-1261-5_3 |  | Less common in residential areas, as compared to right-of-way habitats in urban habitats. Declines in wintering/migrating population use in marshy areas after construction of an apartment complex |
| Zenaida_galapagoensis | Avoid | 0 | 10.1676/05-010.1 |  | Breed on the Galapagos Islands |
| Zonotrichia_atricapilla | Avoid | 0 | 10.1642/AUK-16-27.1 |  | Breed in shrub habitats near tree line in the Canadian Rockies |
| Zonotrichia_capensis | Avoid | 0 | 10.1007/s10336-012-0928-x |  | Abundance is negatively associated with urbanization |
| Passer_domesticus | Exploit | 0 | 10.1525/cond.2008.8409 |  | Abundance is greatly positively associated with urbanization, excluding other species from urban habitats |
| Pycnonotus_goiavier | Exploit | 0 | 10.1016/j.landurbplan.2008.10.013 |  | Highly common in urban areas, outcompetes other species throughout urban area |
